# Automatic segmentation of myocardium at risk in T2-weighted cardiovascular magnetic resonance

**DOI:** 10.1186/1532-429X-14-S1-O20

**Published:** 2012-02-01

**Authors:** Jane Sjogren, Joey F Ubachs, Henrik Engblom, Marcus Carlsson, Hakan Arheden, Einar Heiberg

**Affiliations:** 1 Dept.Clinical Physiology, Skåne University Hospital, Lund University, Lund, Sweden; 2 Dept. Numerical Analysis, Centre for mathematical Sciences, Lund University, Lund, Sweden

## Summary

This study presents a newly developed automatic method for segmentation of myocardium at risk using a priori knowledge on perfusion territories. The new automatic method shows a low bias and high correlation to manual delineation and a bias and correlation closer to inter observer variability for manual delineation than three existing threshold methods, 2SD from remote, FWHM and Otsu.

## Background

T2-weighted cardiovascular magnetic resonance (CMR) has been shown to be a promising technique for determination of ischemic myocardium, referred to as myocardium at risk (MaR), after an acute coronary event. Quantification of MaR in T2-weighted CMR has been proposed to be performed by manual delineation or the threshold methods of two standard deviations from remote (2SD), full width half maximum intensity (FWHM) or Otsu. However, manual delineation is subjective and threshold methods have inherent limitations related to threshold definition and lack of a priori information about cardiac anatomy and physiology. Therefore, the aim of this study was to develop an automatic segmentation algorithm for quantification of MaR using anatomical a priori information.

## Methods

Forty-seven patients with first-time acute ST-elevation myocardial infarction underwent T2-weighted CMR within 1 week after admission. Endocardial and epicardial borders of the left ventricle, as well as the hyper enhanced MaR regions were manually delineated by experienced observers and used as reference method. A new automatic segmentation algorithm, called Segment MaR, defines the MaR region as the continuous region most probable of being MaR, by estimating the intensities of normal myocardium and MaR with an expectation maximization algorithm and restricting the MaR region by an a priori model of the maximal extent for the user defined culprit artery. The segmentation by the new Segment MaR algorithm was compared against inter observer variability of manual delineation and the threshold methods of 2SD, FWHM and Otsu using Bland-Altman bias (mean ± standard deviation) and linear regression analysis (correlation coefficient).

## Results

MaR assessed by manual delineation was 32.9± 10.9 % of LVM and MaR assessed by Segment MaR was 31.0 ± 8.8 %. There was a low bias, -1.9 ± 6.4 % of LVM and strong correlation, R=0.81 when Segment MaR was compared to manual delineation of MaR (Table 1, Figure 1). The inter observer variability of manual delineation was -2.3 ± 4.9 % of LVM. The bias for Segment MaR was lower than for the threshold methods of 2SD, FWHM and Otsu, -7.7 ± 11.4% of LVM, -21.0 ± 9.9% of LVM and 5.3 ± 9.6% of LVM, respectively (Table 1). In Figure 1. MaR as percentage of LVM for Segment MaR (panel A), manual delineation by second observer (panel B) and the threshold method of 2SD (panel C) is plotted against manual delineation by reference observer.

**Table 1:** Comparison of segmentation of myocardium at risk by Segment MaR, manual second observer delineation, 2SD threshold, FWHm threshold and Otsu threshold to manual delineation by reference observer as bias in percentage of left ventricular mass (LVM) and as regression in R-value.

	MaR bias [% of LVM]	Regression R-value
Segment MaR	-1.9 ± 6.4	0.81
Manual second observer delineation	-2.3 ± 4.9	0.91
2SD threshold	-7.7 ± 11.4	0.38
FWHM threshold	-21.0 ± 9.9	0.41
Otsu threshold	5.3 ± 9.6	0.47

## Conclusions

There is a good agreement between automatic Segment MaR and manually assessed MaR in T2-weighted CMR. The Segment MaR method has lower bias and higher correlation than the threshold methods of 2SD, FWHM and Otsu. Thus, the proposed algorithm seems to be a promising, objective method for standardized MaR quantification in T2-weighted CMR.

## Funding

This study has been funded by the Swedish Research Council (2008-2461, 2008-2949), The Swedish Heart and Lung Foundation, The Medical Faculty of Lund University, Sweden, and Region of Scania, Sweden.

